# Supplementary material for: Arabidopsis HAK5 under low K+ availability operates as PMF powered high-affinity K+ transporter
Source: Nat Commun. 2024 Oct 3;15:8558. doi: 10.1038/s41467-024-52963-6 (PMC11450230; doi:10.1038/s41467-024-52963-6)
Supplement: Supplementary file 1 — Supplementary Information [file 41467_2024_52963_MOESM1_ESM.pdf]

Supplementary Information

Supplementary Figures 1-7

Supplementary Tables 1 and 2

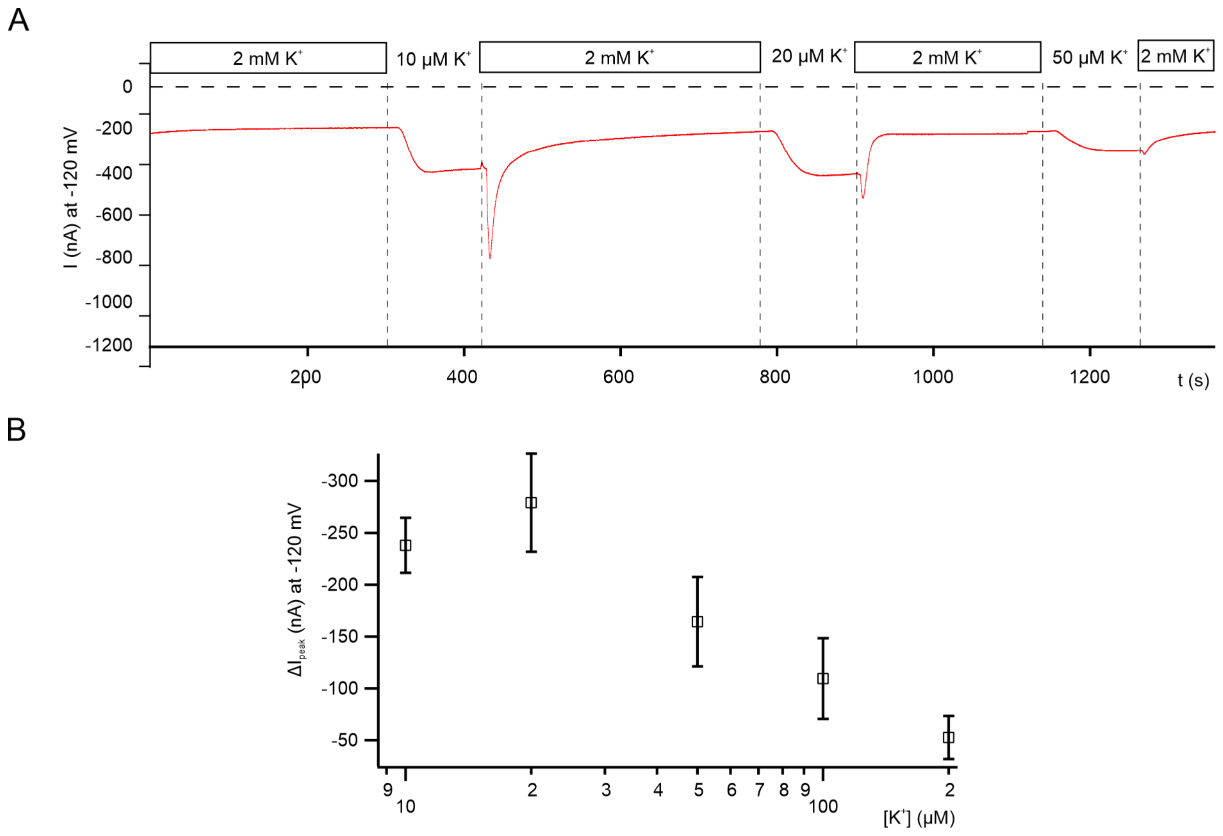

Supplementary Fig. 1: Related to Fig.1, Potassium dependent activation of AtHAK5.

A) Potassium-induced current response at -120 mV of oocytes co-expressing AtHAK5 and CIPK23/CBL1 in the presence of different K^+ concentrations at pH 4.5. Representative current trace is shown. **B)** Whole-oocyte low K^+-induced peak currents (ΔI_{peak}) at -120 mV and pH4.5 from oocytes expressing AtHAK5 with CIPK23/CBL1 plotted against the applied K^+ concentration (n = 4 experiments for 50, 100 and 200 µM K^+, n = 5 experiments for 10 µM K^+, n = 6 experiments for 20 µM K^+, mean ± SD).

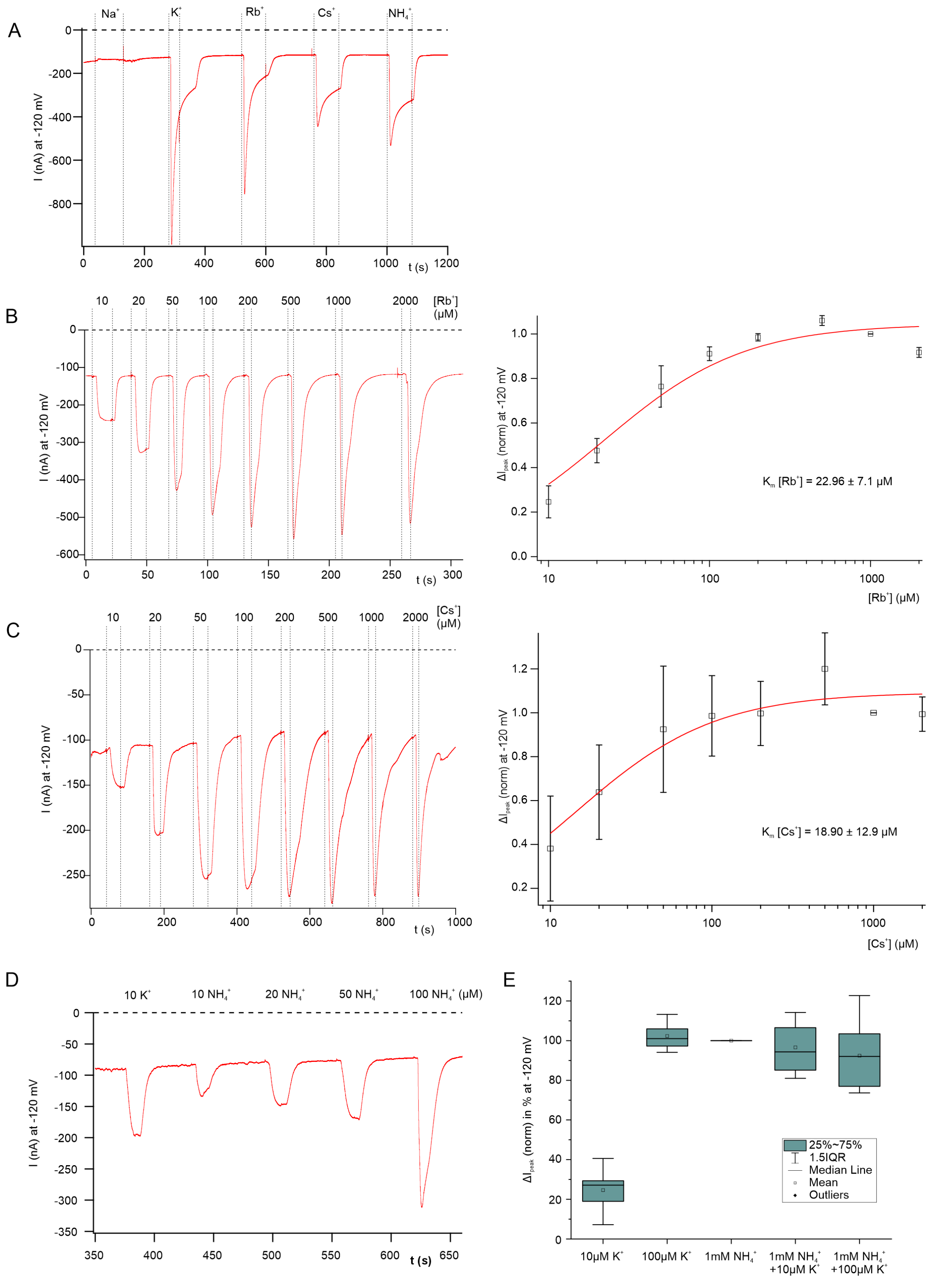

Supplementary Fig. 2: Related to Fig.2, Cation dependency of AtHAK5.

A) Cation-induced current response at -120 mV of oocytes co-expressing AtHAK5 and CIPK23/CBL1 in the presence of either 2 mM Li^+, Na^+, K^+, Rb^+, Cs^+ or NH_4^+. Representative current trace is shown. **B)** Left panel: Representative current response of

AtHAK5/CIPK23/CBL1 co-expressing oocytes upon application of different Rb^+ concentrations. Right panel: Normalized whole-oocyte Rb^+-induced peak currents (ΔI_{peak}) at -120 mV (pH4.5) plotted against the applied Rb^+-concentration. K_m (Rb^+) was calculated by fitting ΔI_{peak} with a Michaelis-Menten equation. (n = 6 experiments, mean $\pm$ SD). **C)** Left panel: Representative current response of AtHAK5/CIPK23/CBL1 co-expressing oocytes upon application of different Cs^+ concentrations. Right panel: Normalized whole-oocyte Cs^+-induced peak currents (ΔI_{peak}) at -120 mV (pH4.5) plotted against the applied Cs^+-concentration. K_m (Cs^+) was calculated by fitting ΔI_{peak} with a Michaelis-Menten equation. (n = 10 experiments, mean $\pm$ SD). **D)** Representative current response of AtHAK5/CIPK23/CBL1 co-expressing oocytes at pH 4.5 upon application of either 10 µM K^+ or different NH_4^+-concentrations as indicated in the figure. **E)** Box plot of normalized whole oocyte peak currents of AtHAK5 and CIPK23/CBL1 co-expressing oocytes at -120 mV in the presence of 10 µM or 100µM K^+ compared to either 1 mM NH_4^+ alone or supplemented with 10 µM or 100 µM K^+, respectively. Currents were normalized to the current response in 1 mM NH_4^+, set to 100 % (n = 5 experiments for 10 µM and 100 µM K^+, n = 7 experiments for 1 mM NH_4^+ alone or supplemented with 10 µM or 100 µM K^+, mean $\pm$ SD).

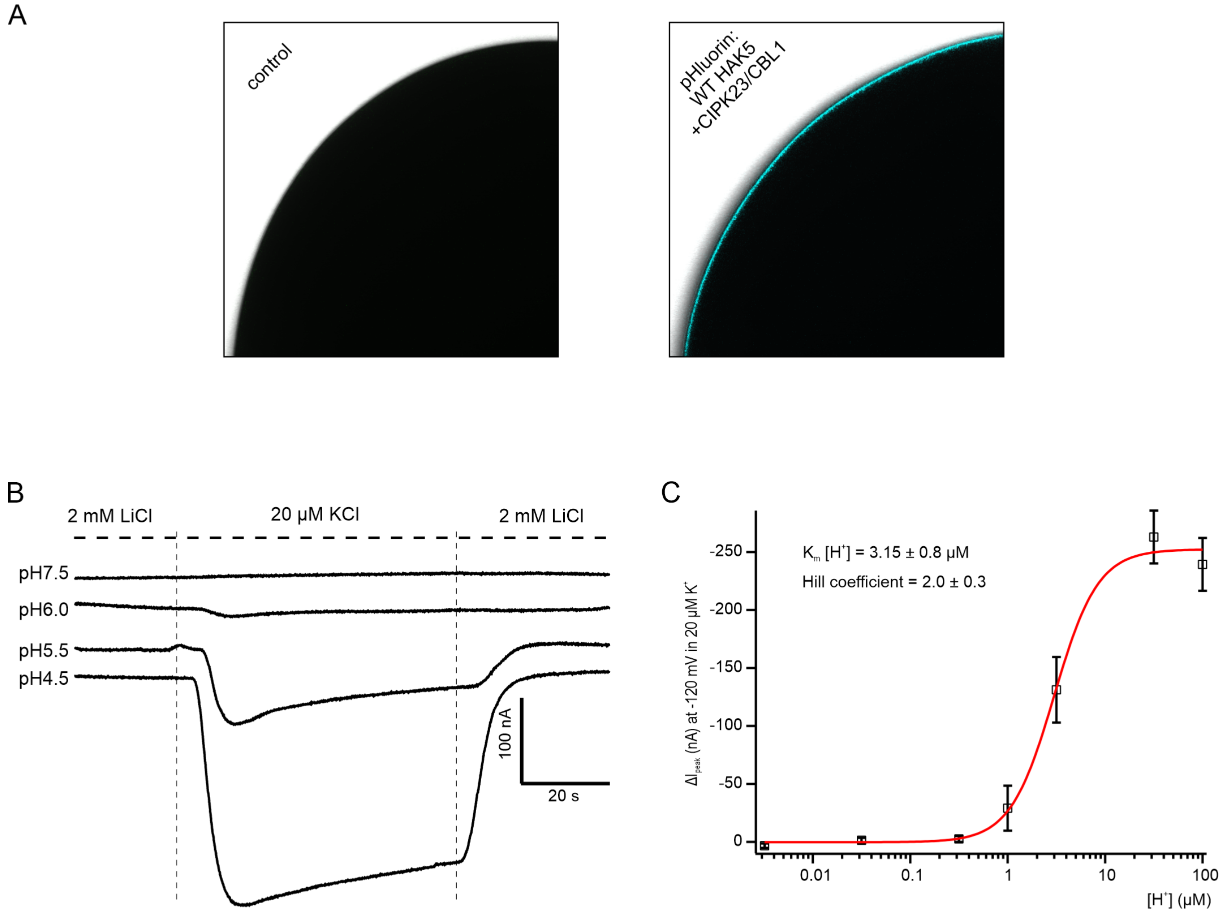

Supplementary Fig. 3: Related to Fig.3, pH-dependency of AtHAK5.

A) Representative image of an oocyte expressing WT HAK5 fused with pHluorin at the N-terminus compared to a control oocyte. Images of one quarter of an oocyte from at least four independent experiments are shown. **B)** Representative current response of AtHAK5/CIPK23/CBL1 co-expressing oocytes upon application of 20µM K^+ at different pH (as indicated in the figure). **C)** Whole-oocyte K^+-induced peak currents (ΔI_{peak}) at -120 mV plotted against the applied H^+-concentration. K_m (H^+) was calculated by fitting ΔI_{peak} with a Hill equation. (n = 4 experiments, mean ± SD).

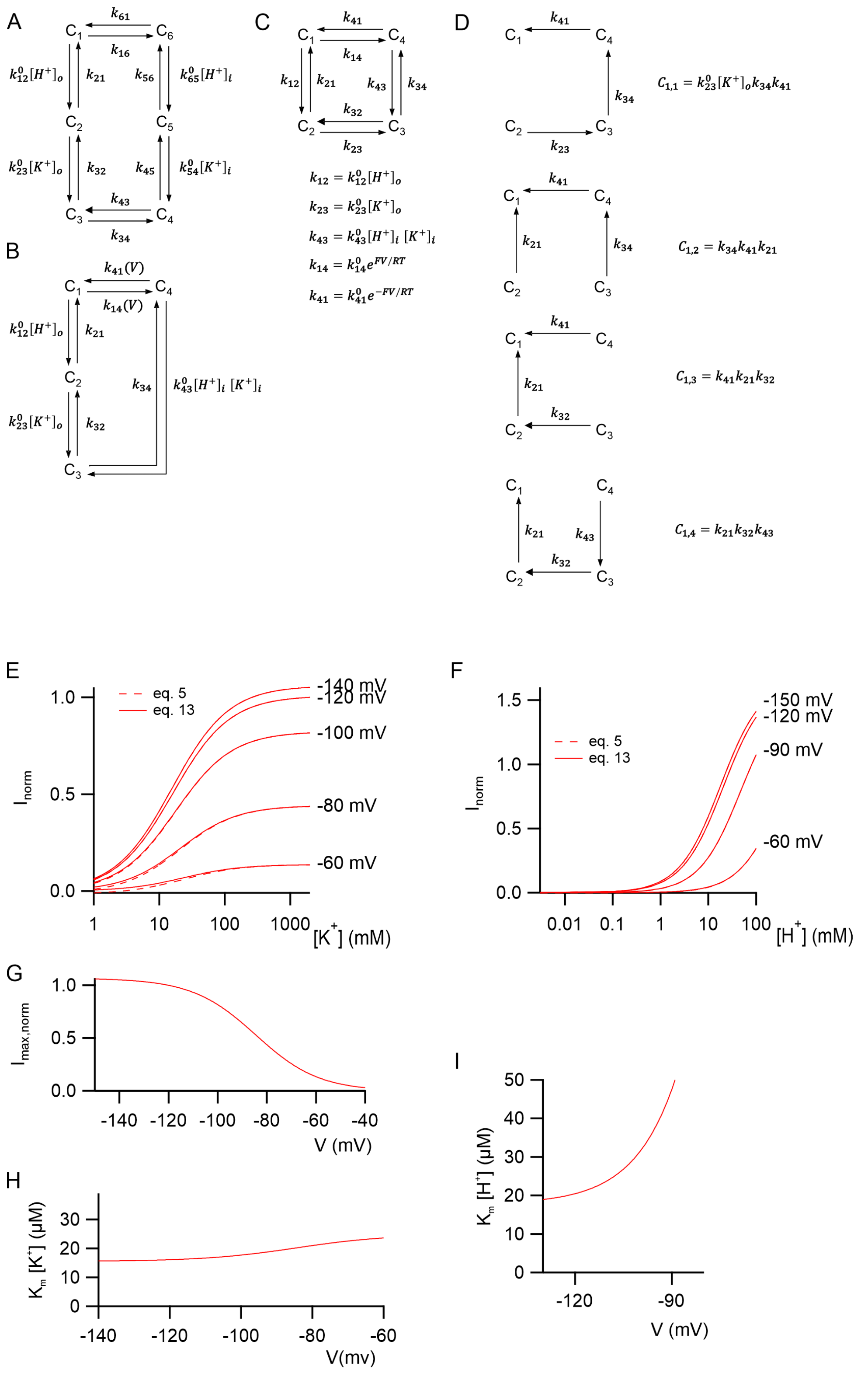

Supplementary Fig. 4: Related to Fig.4, Mathematical modelling of HAK5 transport characteristics.

A) Six state kinetic scheme. **B)** Simplified kinetic model. States 4, 5, and 6 are grouped into state 4. **C)** The same simplified kinetic model as in B written in a compact form. The rate constants dependent on protons, potassium, and membrane voltage are explicitly written

below. **D)** Procedure to find the 16 terms in the denominator in equation (7) in the appendix. The states $C_{1,j}$ with j=1,2,3, and 4 are listed on the right side of the kinetic scheme used to derive them. By applying the same procedure, all the states $C_{i,j}$ can be obtained. **E)** Simulation of currents at different voltages versus external potassium concentrations. Dotted and solid lines were obtained from equation 7 and 15. respectively. The value at 2 mM potassium and -120 mV was used for normalisation. **F)** Peak currents at different voltages versus external proton concentrations. Dotted and solid lines were obtained from equation 7 and 15 and are indistinguishable. Normalized currents using the value at pH 4.5 and -120 mV. **G)** Maximal current for external potassium versus applied voltage. Current was normalized using the value at -120 mV. **H)** Apparent affinity for external potassium versus applied voltage. **I)** Apparent affinity for external protons versus applied voltage.

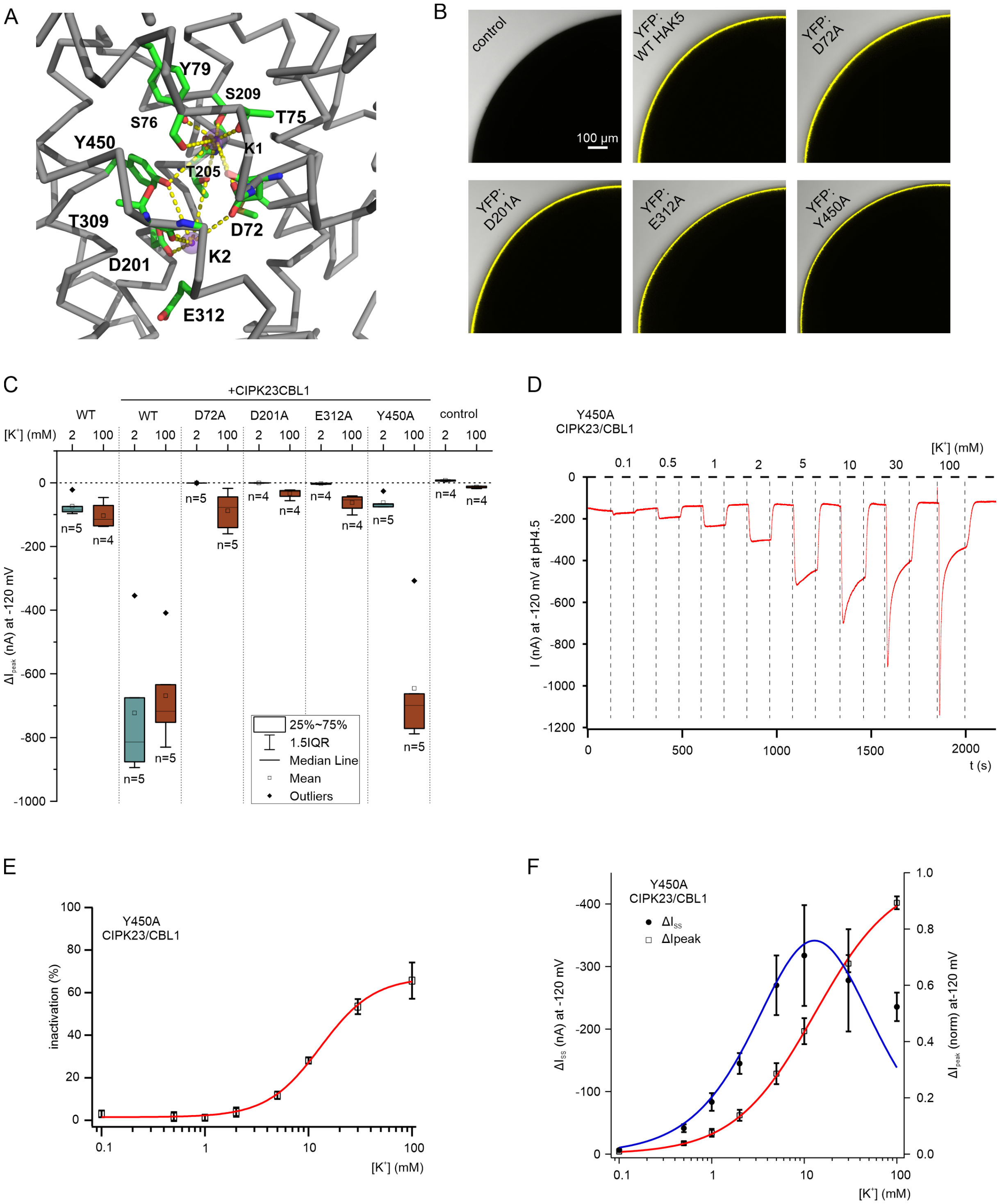

Supplementary Fig. 5: Related to Fig.6, Molecular nature of K^+ sensing.

A) Magnification of Fig. 6A showing details of the two potassium coordination sites. The potassium ion in the upper coordination site (closer to the extracellular pore entry) is labeled with K1 and is coordinated by residues D72, T75, S76, Y79, S209 and Y450. The potassium ion in the lower coordination site (closer to the intracellular pore entry) is labeled with K2. The latter K^+ ion is coordinated by fewer residues, namely D72, D201, T205, T309 and Y450. **B)**

Images of oocytes expressing either WT HAK5 or the mutants D72A, D201A E312A, Y450A fused with a YFP at the N-terminus. Representative images of one quarter of an oocyte from at least four independent experiments are shown. **C)** Box blot of K^+-induced peak currents (ΔI_{peak}) at -120 mV of oocytes expressing either WT AtHAK5 alone or WT AtHAK5, mutant D72A, D201A, E312A or Y450A in the presence of CIPK23/CBL1 when challenged with either 2 mM or 100 mM K^+. Water-injected oocytes were used as control (number of experiments is indicated in the figure). **D)** Representative current trace of oocytes co-expressing AtHAK5 Y450A and CIPK23/CBL1 at -120 mV and pH4.5 when challenged with different K^+ concentrations (as indicated in the figure). **E)** Degree of inactivation (in %) derived from similar experiments as shown in D) were plotted against the applied K^+ concentration (n = 5 experiments, mean $\pm$ SD). **F)** Normalized whole-oocyte K^+-induced peak currents (ΔI_{peak}) or steady-state currents (ΔI_{SS}) at -120 mV at pH4.5 are plotted against the applied K^+-concentration. K_m (K^+) was calculated by fitting ΔI_{peak} with a Michaelis-Menten equation. The modified Michaelis-Menten function used to fit ΔI_{SS} is described in the methods section (n = 5 experiments $\pm$ SD).

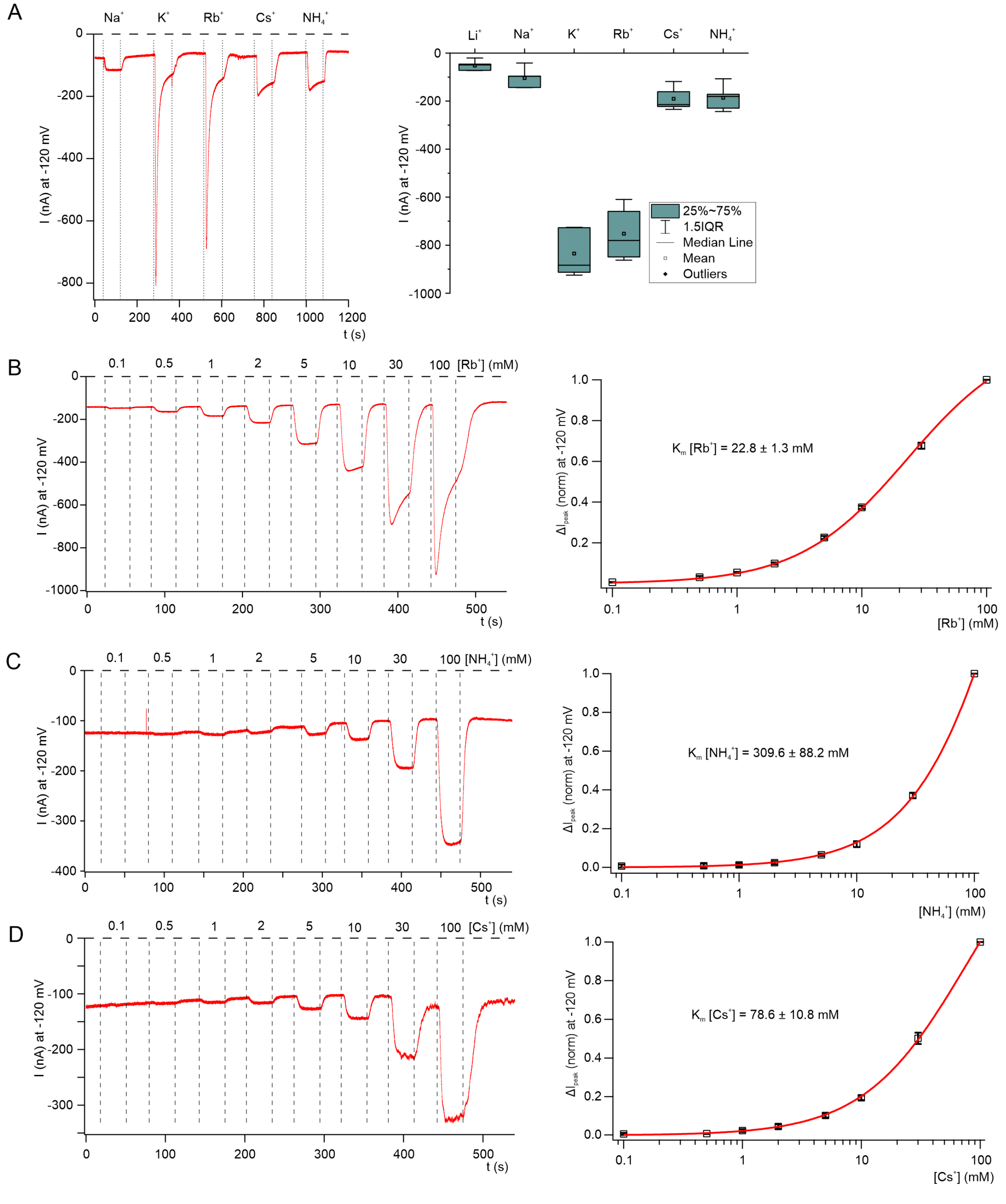

Supplementary Fig. 6: Related to Fig.6, Cation dependency of AtHAK5 mutant Y450A.

A) Cation-induced currents response at -120 mV of oocytes co-expressing AtHAK5 mutant Y450A and CIPK23/CBL1 in the presence of either 100 mM Li^+, Na^+, K^+, Rb^+, Cs^+ or NH_4^+. Left panel: Representative current trace is shown. Right panel: Box plot of cation-induced currents at -120 mV (n = 5 experiments). **B)** Left panel: Representative current response of AtHAK5 Y450A/CIPK23/CBL1 co-expressing oocytes upon application of different Rb^+ concentrations. Right panel: Normalized whole-oocyte Rb^+-induced peak currents (ΔI_{peak}) at -120 mV (pH4.5)

plotted against the applied Rb^+-concentration. K_m (Rb^+) was calculated by fitting ΔI_{peak} with a Michaelis-Menten equation. (n = 5 experiments, mean ± SD). **C)** Left panel: Representative current response of AtHAK5 Y450A/CIPK23/CBL1 co-expressing oocytes upon application of different NH_4^+ concentrations. Right panel: Normalized whole-oocyte NH_4^+-induced peak currents (ΔI_{peak}) at -120 mV (pH4.5) plotted against the applied NH_4^+-concentration. K_m (NH_4^+) was calculated by fitting ΔI_{peak} with a Michaelis-Menten equation. (n = 4 experiments, mean ± SD). **D)** Left panel: Representative current response of AtHAK5 Y450A/CIPK23/CBL1 co-expressing oocytes upon application of different Cs^+ concentrations. Right panel: Normalized whole-oocyte Cs^+-induced peak currents (ΔI_{peak}) at -120 mV (pH4.5) plotted against the applied Cs^+-concentration. K_m (Cs^+) was calculated by fitting ΔI_{peak} with a Michaelis-Menten equation. (n = 5 experiments, mean ± SD).

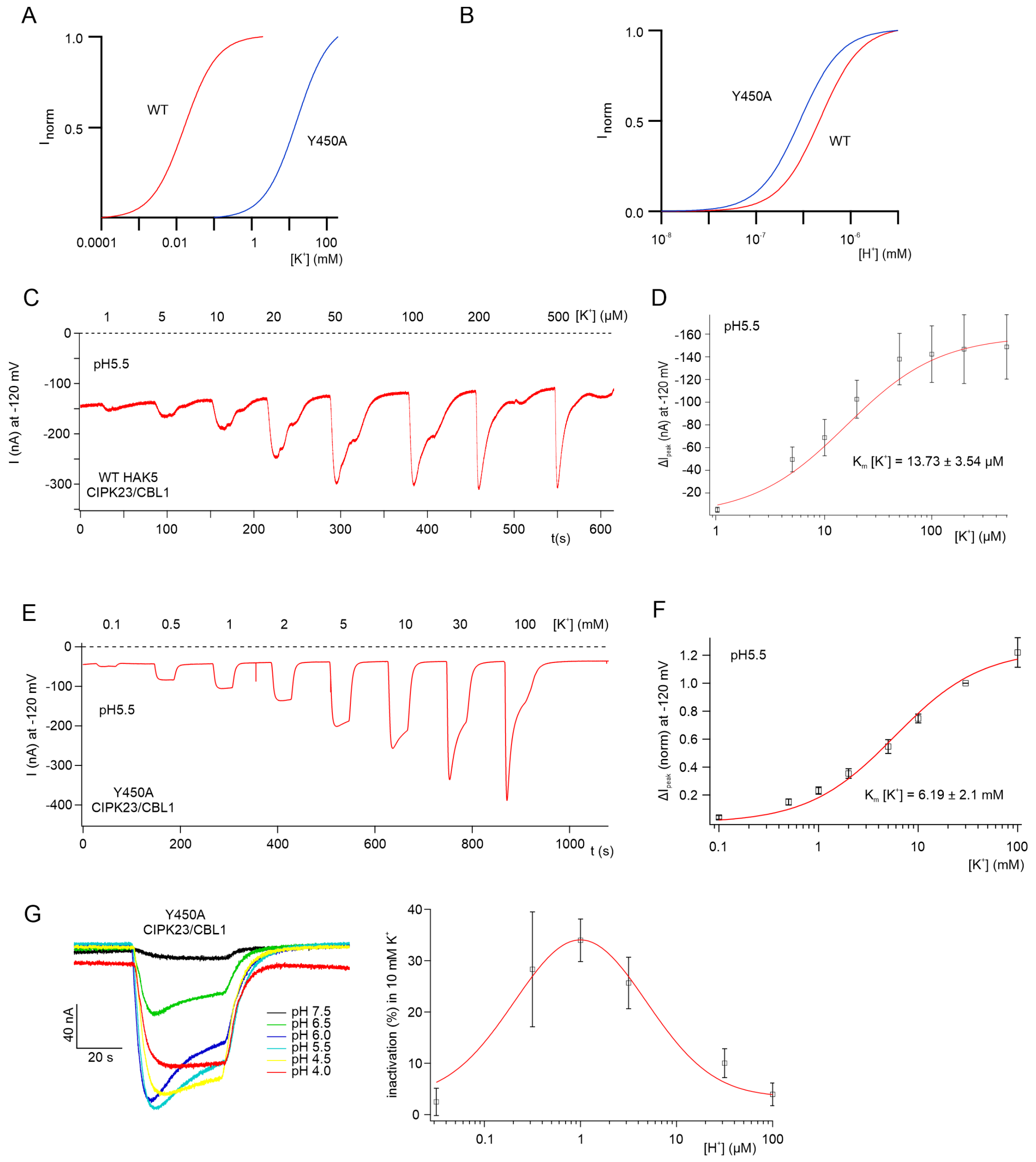

Supplementary Fig. 7: Related to Fig.6, Molecular nature of K^+ sensing.

A) Simulation of currents at -120 mV from wild-type transporter (WT) and mutant (Y450) versus external potassium concentrations. Currents were normalized using the value at 2 mM for WT and at 200 mM for Y450A. **B)** Simulation of currents at -120 mV from wild-type transporter (WT) and mutant (Y450) versus external proton concentrations. The value at pH 3 was used to normalise currents. **C)** Representative current trace of an oocyte co-expressing AtHAK5 and CIPK23/CBL1 at -120 mV and pH5.5 when challenged with different K^+ concentrations (as indicated in the figure). **D)** Whole-oocyte K^+-induced peak currents (ΔI_{peak})

at -120 mV and pH5.5 from oocytes expressing AtHAK5 with CIPK23/CBL1 plotted against the applied K^+-concentration (n = 3 experiments for 500 µM, n = 4 experiments for all other concentrations, mean ± SD). K_m [K^+] was calculated by fitting ΔI with a Michaelis-Menten equation. (n = 6 experiments, mean ± SD).

E) Representative current trace of oocytes co-expressing AtHAK5 Y450A and CIPK23/CBL1 at -120 mV and pH5.5 when challenged with different K^+ concentrations (as indicated in the figure). **F)** Normalized whole-oocyte K^+-induced peak currents (ΔI_{peak}) at -120 mV and pH5.5 from oocytes expressing the mutant Y450A with CIPK23/CBL1 plotted against the applied K^+-concentration. K_m [K^+] was calculated by fitting ΔI_{peak} with a Michaelis-Menten equation. (n = 4 experiments, mean ± SD). **G)** Left panel: Representative superimposed current responses of AtHAK5 mutant Y450A/CIPK23/CBL1 co-expressing oocytes upon application of 10 mM K^+ at different pH (as indicated in the figure). Right panel: Percentage of inactivation at 10 mM K^+ plotted against the H^+ concentration (n = 3 experiments for pH7.5, n = 5 experiments for pH6.5, pH6, pH5.5, pH4.5 and pH4, mean ± SD).

Supplementary Table 1

Numerical values assigned to the rate constants in the four-state model proposed for the wild-type transporter (WT) and the mutant (Y450A). The rate constant k_{12}, obtained by equation (5) as a consequence of the principle of microscopic reversibility, was 10^{-3} and 10^{-6} s^{-1} respectively for wild-type and mutant transporter.

**rate constant**	**WT**	**Y450A**
k_{41}^{0}	5 s^{-1}	5 s^{-1}
k_{12}^{0}	5 10^{3} s^{-1} M^{-1}	5 10^{3} s^{-1} M^{-1}
k_{23}^{0}	10^{6} s^{-1} M^{-1}	**10^{3} s^{-1} M^{-1}**
k_{34}	0.1	0.1
k_{14}^{0}	100	100
k_{43}^{0}	10^{9} s^{-1} M^{-2}	10^{9} s^{-1} M^{-2}
k_{32}	25	25

Supplementary Table 2

List of primers used for cloning and mutagenesis

**primer name**	**primer sequence**
AtHAK5 u fwd	GGCTTAAUATGGATGGTGAGGAACATCAAATAG
AtHAK5 u rev	GGTTTAAUCCTAACTCATAGGTCATGCCAACC
AtHAK5 u D72A fwd	ATGGAGCTAUCGGGACATCGCCATTATAT
AtHAK5 u D72A rev	ATAGCTCCAUACACCACTCCTAGGCTCT
AtHAK5 u D201A fwd	ATTGGTGCUGGAATCCTTACTCCTTCCATCTC
AtHAK5 u D201A rev	AGCACCAAUGACCATGGAAGTTC
AtHAK5 u E312A fwd	ACGGCGGCAAUGTTTGCTGATCTAGGT
AtHAK5 u E312A rev	ATTGCCGCCGUCCCAGTGATGCAGAG
AtHAK5 u Y450A fwd	ACGCTGCTGGGAUCGCCGTTGTAACC
AtHAK5 u Y450A rev	ATCCCAGCAGCGUGACCAATCTTCTCAGTAGT
